# Screening COVID-19 by Swaasa AI platform using cough sounds: a cross-sectional study

**DOI:** 10.1038/s41598-023-45104-4

**Published:** 2023-10-25

**Authors:** Padmalatha Pentakota, Gowrisree Rudraraju, Narayana Rao Sripada, Baswaraj Mamidgi, Charishma Gottipulla, Charan Jalukuru, Shubha Deepti Palreddy, Nikhil Kumar Reddy Bhoge, Priyanka Firmal, Venkat Yechuri, Manmohan Jain, Venkata Sudhakar Peddireddi, Devi Madhavi Bhimarasetty, S. Sreenivas, Kesava Lakshmi Prasad K, Niranjan Joshi, Shibu Vijayan, Sanchit Turaga, Vardhan Avasarala

**Affiliations:** 1 Andhra Medical College, Visakhapatnam, India; 2 Salcit Technologies, Jayabheri Silicon Towers, Hyderabad, India; 3 C-CAMP, Bangalore, India; 4 Qure.Ai Technologies, Oberoi Commerz II, Mumbai, India; 5 NDORMS, University of Oxford, Oxford, UK; 6 Otolaryngology - Head and Neck Surgery, Northeast Ohio Medical University, Rootstown, USA

**Keywords:** Infectious diseases, Health care

## Abstract

The Advent of Artificial Intelligence (AI) has led to the use of auditory data for detecting various diseases, including COVID-19. SARS-CoV-2 infection has claimed more than six million lives to date and therefore, needs a robust screening technique to control the disease spread. In the present study we created and validated the Swaasa AI platform, which uses the signature cough sound and symptoms presented by patients to screen and prioritize COVID-19 patients. We collected cough data from 234 COVID-19 suspects to validate our Convolutional Neural Network (CNN) architecture and Feedforward Artificial Neural Network (FFANN) (tabular features) based algorithm. The final output from both models was combined to predict the likelihood of having the disease. During the clinical validation phase, our model showed a 75.54% accuracy rate in detecting the likely presence of COVID-19, with 95.45% sensitivity and 73.46% specificity. We conducted pilot testing on 183 presumptive COVID subjects, of which 58 were truly COVID-19 positive, resulting in a Positive Predictive Value of 70.73%. Due to the high cost and technical expertise required for currently available rapid screening methods, there is a need for a cost-effective and remote monitoring tool that can serve as a preliminary screening method for potential COVID-19 subjects. Therefore, Swaasa would be highly beneficial in detecting the disease and could have a significant impact in reducing its spread.

## Introduction

The SARS-CoV-2 infection first surfaced at the end of December 2019, affecting nearly 769 million people across the globe^1^. The virus transmission begins once a healthy individual is exposed to the respiratory droplets originating from an infected person. The average incubation period for the disease symptoms to manifest varies from 2–14 days^2^. The early symptoms comprise dry cough, fever, fatigue, and loss of smell and taste. Some patients may experience shortness of breath, cardiac issues, and pneumonia-like symptoms, which can ultimately result in death. Many people are also experiencing post-covid acute symptoms which affect their overall health status^3,4^. The containment of COVID-19 outbreaks became very difficult because of the unavailability of quick and effective pre-screening techniques. Most of the viral and serological testing methods available are very expensive, time-consuming, require technical expertise and are not always reliable, especially in detecting the new SARC-CoV-2 variants^5,6^.

Cough is a common symptom of COVID-19^7^. Physiologically, coughing is responsible for removing any obstruction in the airways via explosive expulsion of the air^8^. While this serves some benefit to the patient, it often results in the dissemination of airborne infectious aerosols^9,10^. That being said, cough is very useful in diagnosing and detecting various disease processes. Literature suggests that glottis movement during a cough can be used to differentiate between various respiratory conditions including pertussis, bronchitis and asthma^11,12^. With cough being such a prominent and unique aspect of multiple disease processes, more reports suggest its possible use in detecting Covid-19^13,14^. Cough sound analysis has proven effective for diagnosing diseases like tuberculosis (TB). However, the application of AI cough analysis for COVID-19 detection has not been fully explored. Further research and validation are necessary to ascertain its accuracy and potential usefulness in the screening of COVID-19.

In the past few years, substantial strides have been made exploring the utility of AI in medicine—particularly in using machine learning techniques to analyse cough sounds for multiple respiratory pathologies^15,16^. In fact, various groups have highlighted the benefits of these machine-learning techniques in detecting COVID-19 over pre-screening methods such as, RT-PCR^17–21^. While there is significant interest and investigation in developing an AI-based model for Covid-19 detection, no current tools are available in the market to our knowledge. This is largely due to the difficulty in the acquisition of cough sound data from crowdsource open access datasets and lack of proper technical/clinical validations to scale up these tools for mass screening of COVID subjects^22,23^.

Our research provides a comprehensive methodology for developing, validating, and testing the “Swaasa AI platform” as a rapid Point of Care tool to screen and prioritize potential COVID-19 cases. This Software as a Medical Device (SaMD) evaluates SARS-CoV-2 infection through analysis of a 10-s cough sound recording. Our study is distinct in that it includes cough recordings from COVID-19 positive patients, healthy subjects, and patients with various respiratory conditions. We conducted a feature analysis of known COVID-19 and non-COVID-19 coughs and discovered a distinct signature in COVID-19 coughs that a machine learning model can identify. Unlike prior research that relied on crowdsource cough databases for data collection, this study uses data acquired in a clinical setting from a diverse and comprehensive background bolstering its utility as a robust model. We trained two parallel models, a CNN model along with a FFANN model and merged their final layers to increase accuracy. Our model achieved 96% accuracy on the test dataset during derivation and 76% during validation phase, with a positive prediction value of 70.73% in real-time scenarios. This cost-effective, non-invasive screening tool is valuable for detecting COVID-19 cases. However, further validation studies involving larger and more diverse populations are necessary to improve accuracy and applicability globally.

## Materials and methods

### Sample size estimation

To determine the sample size for our study, we employed a formula that incorporates various assumptions about key variables. The formula, n = Z^2^*P(1 − P)/d^2^, involved selecting values for Z (level of confidence), P (anticipated prevalence), and d (precision corresponding to the effect size)^24^. According to the sample size calculation, a total of 1152 subjects was appropriate for validating if the device could detect COVID-19 with a 90% sensitivity on considering a 2.5% error for a 95% confidence interval (CI) and a prevalence of 0.75%. Considering all the conditions, we pooled the data collected from two individual clinical trial studies for developing and evaluating our model. We considered a total of 1052 participants in the present study, out of which 62% were controls. Control subjects comprise healthy individuals as well as subjects who were displaying various respiratory disease symptoms but were negative for COVID-19 via RT-PCR. To prevent any potential bias in the model, we ensured that an equal number of COVID-19 and non-COVID-19 subjects were used during the training process.

### Data collection

The cough data was collected at Andhra Medical College, Visakhapatnam, India as a part of four individual studies entitled “Development, Validation, Pilot Deployment of an ultra-scalable technology—Swaasa AI, as an auxiliary to COVID-19 Rapid test” and “COVID-19 Cough Sound Analysis Using Swaasa Artificial Intelligence Platform”. The studies were registered under Clinical Trials Registry- India CTRI/2021/09/036,489, CTRI/2021/07/035,096, and were begun after getting the approval from the Andhra Medical College—Institutional Ethics Committee (IEC). The methodologies performed throughout the study were in accordance with the set guidelines. A duly signed written informed consent was also collected from all the enrolled subjects before starting the trial, which was followed by collection of the demographic details and the vital signs. Patients were then interviewed for the Part I of the St. George's Respiratory Questionnaire (SGRQ) and COVID-19 symptoms in order to gather their symptoms^25^. Next, the cough sound was collected by a trained health care personnel using a smartphone (Android or iPhone). The participants were instructed to take a deep breath and cough 2–3 times until the recording stopped i.e., 10 s. We also provided specific instructions to each participant, including sitting comfortably in a quiet place, holding the recording device 4–8 inches away from their mouth at 90-degree angle with their face. We also applied a noise reduction algorithm to filter out background noise in the audio recordings. Valid coughs were detected using a cough/non-cough classifier, which screened the dataset for coughs with high background noise. We also implemented several safety measures to prevent the transmission of disease, including requiring participants to wear a surgical mask during the audio recording process and cleaning the phone used for recording after each recording.

Following the cough sample collection, the patients were subjected to a reference standard test (RT-PCR). The inclusion criteria for the enrolment are that the Patients must be (a) male and female patients age ≥ 18 years, who were (b) recently diagnosed with COVID-19 (for validation phase only) and were (c) able to read, understand and sign the informed consent form. Whereas male and female patients age < 18 years, who were (b) on ventilators support, (c) asymptomatic patients attending isolation ward for COVID-19 testing and (d) Pregnant females were completely excluded from the current study. COVID-19 precautionary and infection control measures were followed strictly.

### Model development and training

**Figure 1:**
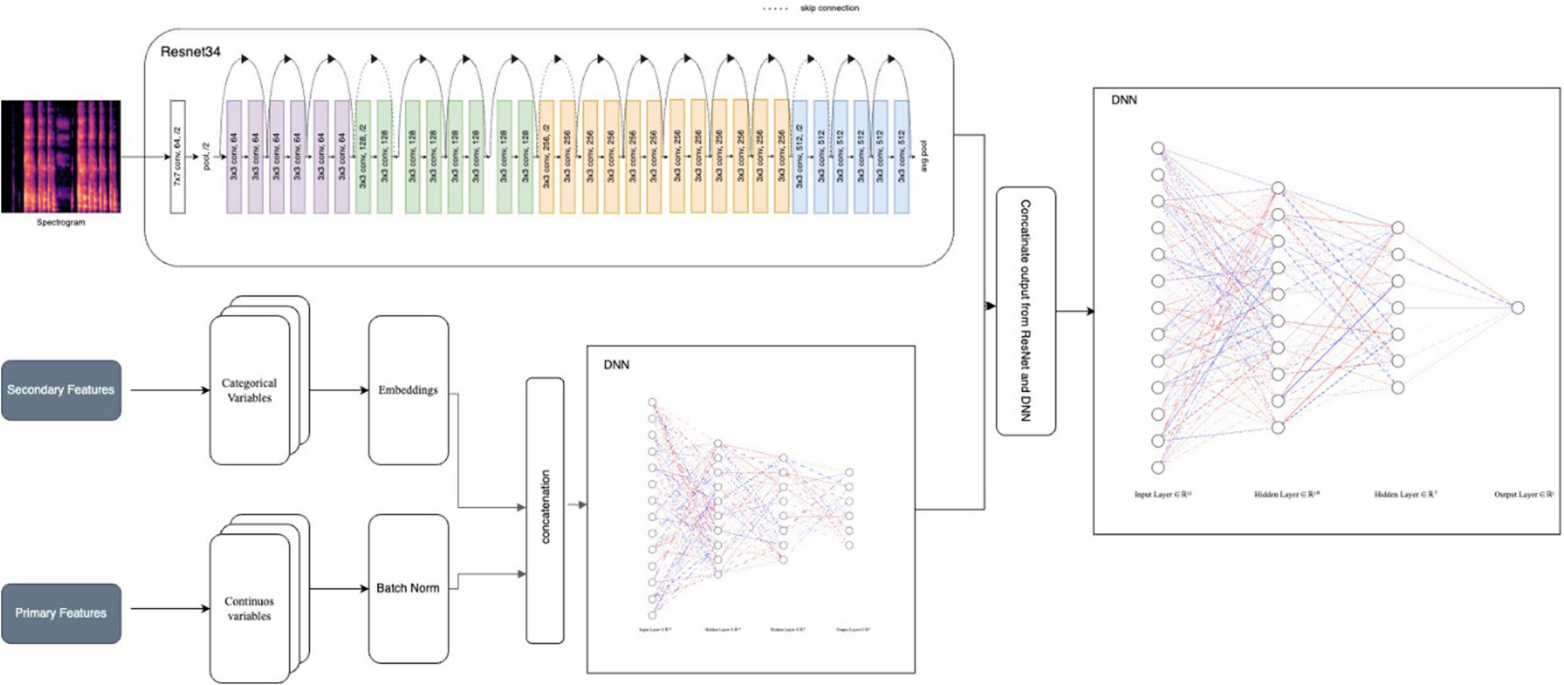
Block Diagram illustrating the flow of the COVID-19 prediction model.

In the derivation phase of the study, a machine learning-based model was developed and trained for the detection of COVID-19 using cough sounds. A total of 803 cough events were extracted from cough data collected form 252 subjects which were tested positive for COVID-19 by RT-PCR. Event extraction was carried out using the moving window signal standard deviation technique, and a cough/non-cough classifier was used to segregate the events into actual coughs and non-coughs. A total of 1946 cough events were extracted from both COVID-19 likely-Yes and No subjects. The features were extracted from both the time and frequency domain of each cough event, resulting in 209 features that includes information about age, gender, and various audio features such as Mel Frequency Cepstral Coefficients (MFCC), spectral features (spectral centroid, spectral roll-off, etc.), chroma features, contrast features, tonnentz features, zero-crossing rate (ZCR), energy, skewness, and kurtosis^29^. Correlation-based feature selection was used to reduce the feature size from 209 to 170^26^. When the model is unsure if COVID-19 will be detected as yes/no, it provides an inconclusive output as shown in the block diagram Fig. 1.

### Clinical validation of the model

In the Clinical validation phase, the trained model was tested on 233 subjects recruited from different locations, including isolation wards and COVID testing centers. The subjects underwent both the screening test using the model and the standard RT-PCR test for COVID-19 diagnosis. The test results from both methods were compared to each other by a statistician. A consolidated test summary sheet was generated containing the results of both the standard diagnostic testing and the model's output.

### External validation of the model

During the Pilot Phase, the trained model was externally validated to determine its effectiveness as a screening tool for detecting COVID-19 prior to clinical diagnosis. The sample size for this phase consisted of 177 individuals who were identified as presumptive COVID-19 cases and recruited from a peripheral healthcare center. To measure the model's effectiveness, the ratio of patients truly diagnosed as COVID-19 positive via standard diagnostic techniques to all those who were predicted to be COVID-19 positive via the model was calculated. The diagnostic performance of the model was evaluated using various metrics, including sensitivity, specificity, positive predictive value (PPV), negative predictive value (NPV), and accuracy. Again, the effectiveness of the model was assessed by comparing it with standard diagnostic methods like RT-PCR, and the results were analysed using statistical methods.

### LIME representation

The green portion of the Local interpretable model-agnostic explanations (LIME) representation^27^ illustrates instances in which the model responded positively to a given class, whereas the red portion highlights instances in which it responded negatively. To “explain” a prediction, we refer to the display of textual or visual artefacts that give qualitative understanding of the link between the instance's components (such as words in text, patches in a picture, etc.) and the prediction made by the model.

### Statistical significance

A comprehensive assessment of model performance on the test set includes accuracy, sensitivity, specificity, positive predictive value (PPV), negative predictive value (NPV), and ROC. To measure the variability of these parameters, we used the Clopper-Pearson method^28^ with 95% confidence intervals. To better understand the model's performance in screening COVID-19 subjects, we also calculated the confusion matrix across the test set.

## Results

### Performance parameters in model derivation phase

**Figure 2:**
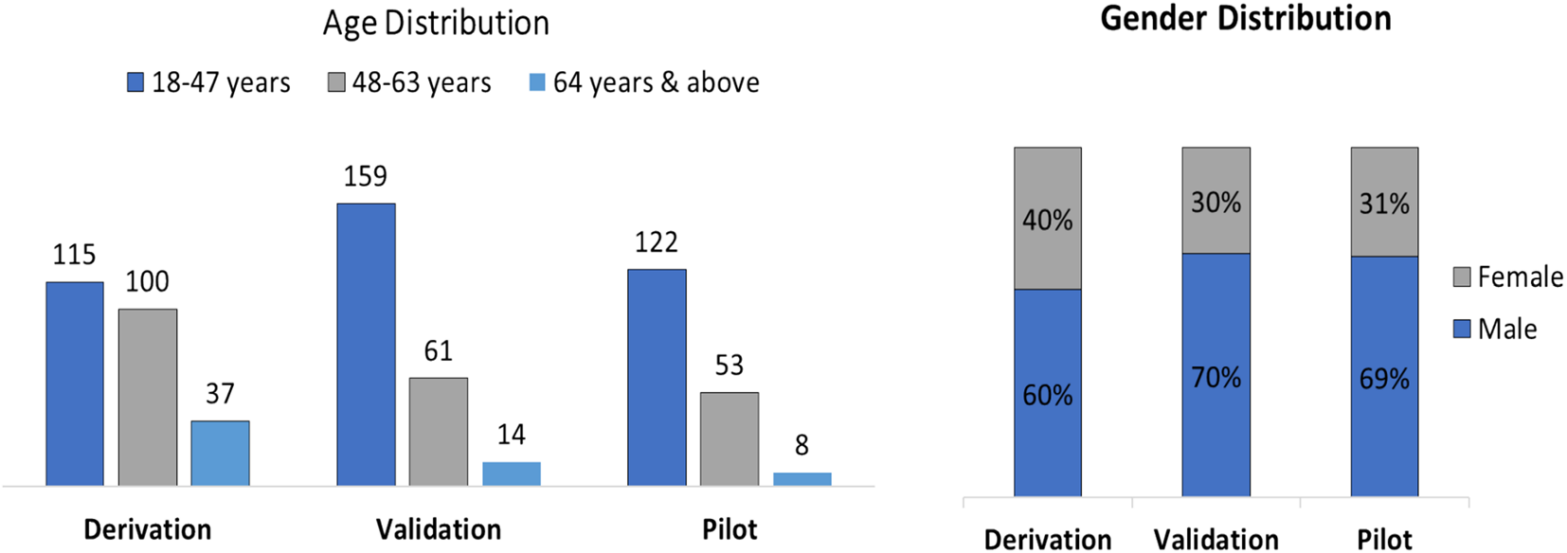
Data distribution in the derivation phase, validation phase and pilot testing.

Cough sound data was collected from 252 COVID-19 positive subjects in the derivation phase. Data collected from 390 COVID-19 negative subjects in one of our earlier studies was also considered in this phase. Among 252 subjects, 60% were male and 40% were female, with age ranging from 18 years to 64 & above (Fig. 2). Subjects were confirmed with COVID-19 by standard diagnosis methods. In this phase multiple data points were collected from the subjects. Each data point was called a record. A total of 803 cough records were collected from 252 patients.

**Figure 3:**
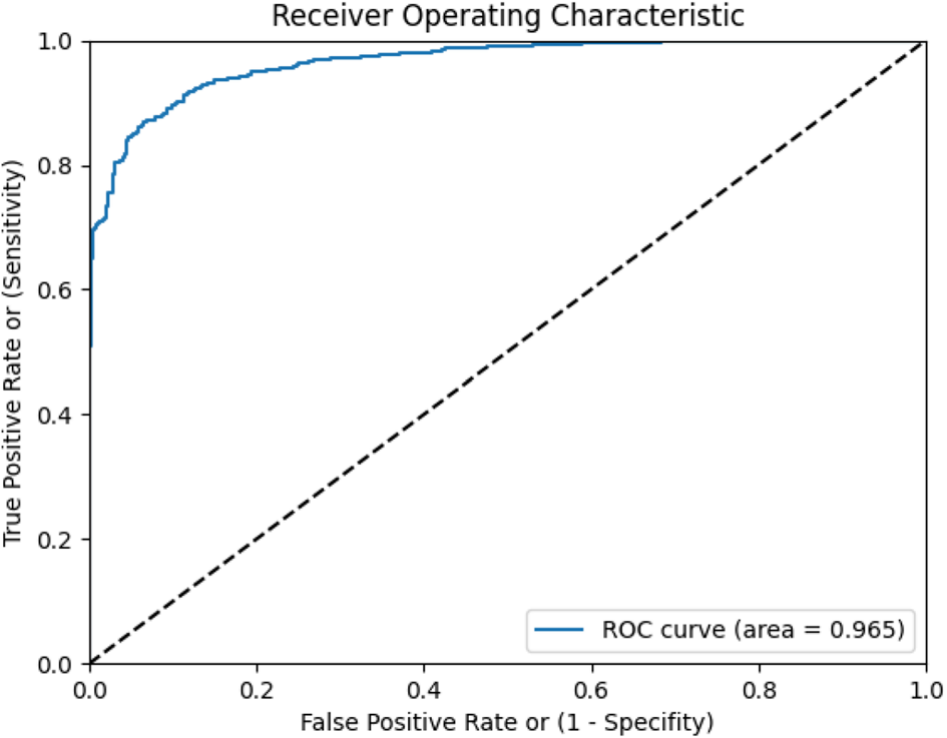
The representative graph for ROC curve, best among tenfold validation of COVID-19 prediction model built using derivation data.

The 252 subject data was divided into training (173) and test (79). The training data was internally divided into training and validation as required to build as well as optimize the model performance based on K-fold cross validation technique. All the 252 subject data was annotated with disease condition as COVID-19 i.e., COVID-19 likely as “yes”. For COVID unlikely, data representing other disease conditions was added from pre-existing datasets^29^ (collected part of earlier studies) in various propositions. A total of 1213 records data was added to various classifiers. The final confusion matrix for derivation phase is represented in Table 1. The performance parameters such as accuracy, sensitivity, specificity, and AUC (Area Under the ROC Curve) of the model in the derivation phase are enlisted in Table 2. The model's ability to produce accurate predictions is extremely effective, as shown by the AUC score of 0.965. The ROC curve in Fig. 3 illustrates the best-performing fold among the ten cross-validation folds.

**Table 1:** Final Confusion matrix for the derivation phase. The values represented in the table are the cough records.

		Actual values (ground truths)		Total
		COVID likely—YES	COVID likely—NO	
Predicted values	COVID likely—YES	256 (TP)	12 (FP)	268
	COVID likely—NO	11 (FN)	278 (TN)	289
	Total	267	290	557

**Table 2:** Performance metrics of the derivation phase.

Accuracy	96%
Sensitivity	95.8%
Specificity	95.6%
AUC	0.95

The model was also evaluated on crowdsourced data, which includes COVID-19 Likely “yes” data from EPFL and Cambridge datasets, whereas COVID-19 Likely “no” is the in-house data. The final confusion matrix for this dataset is presented in Table 3. The performance parameters for the same are listed in Table 4.

**Table 3:** Final Confusion matrix for the Crowdsource (test) data during derivation phase. The values represented in the table are the cough records.

		Actual values (ground truths)		Total
		COVID likely—YES	COVID likely—NO	
Predicted values	COVID likely—YES	527 (TP)	57 (FP)	584
	COVID likely—NO	91 (FN)	443 (TN)	534
	Total	618	500	1118

**Table 4:** Performance metrics of Crowdsource (test) data during derivation phase.

Accuracy	86%
Sensitivity	85%
Specificity	88%
AUC	0.855

The features listed in Table 5 depicts the mean value of the features extracted from individual frames, where we have considered normal as well as respiratory diseases data other than COVID from our previous validation study conducted at Apollo Hospitals, Hyderabad.

**Table 5:** Table showing mean values of the Zero crossing rate (ZCR), spectral centroid and dominant frequency of various respiratory disease conditions, including COVID-19.

Disease conditions	ZCR mean values	Spectral centroid mean values	Dominant frequency mean values
Normal	0.168	2249	844
ILD	0.099	2053	436
COPD	0.08	1947	393
Asthma	0.112	2093	528
Pneumonia	0.118	2249	546
COVID-19	0.246	3710	1287
COVID-19 (Low severity)	0.203	3300	891

### LIME data comparison

Extremely low spectral frequencies have been observed in conditions such as COPD and ILD as compared to asthma, which has an intermediate spectral frequency. On the other hand, we found that the spectral components are very high in diseases in which mucus accumulates in the airways and fluid accumulates in the parenchyma region. High spectral content is the distinguishing feature of COVID-19 cough from other respiratory diseases.

Feature analysis studies of cough sounds have revealed that it could be utilized for distinguishing diseases. The cough duration and frequency distribution has been found to be unique in a specific respiratory disease, including COVID-19^30,31^.

We compared the LIME maps of various respiratory diseases with COVID-19 which are enlisted in Table 6. It can be seen in the maps that each disease has a unique frequency distribution. Green patches were more dominant for COVID-19 in high frequency regions. Whereas LIME maps for normal subjects were reacting negatively even though it has some green patches present. Similarly, pneumonia maps were also present in the high frequency range but has a stronger predominance in the medium frequency range. Even for Asthma most of the dominant green patches were seen in the medium frequency region.

**Table 6:**
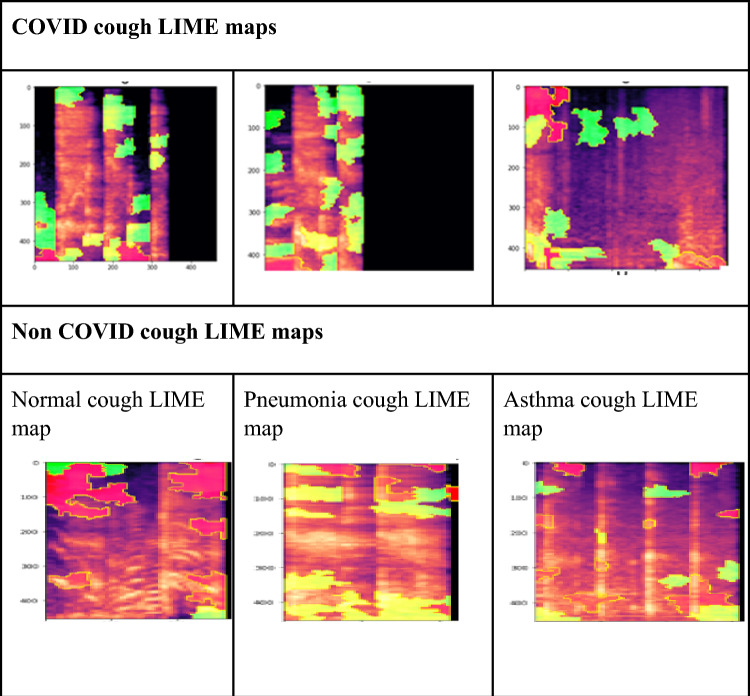
List of different respiratory diseases showing characteristic cough signature, cough spectrograms and related LIME maps.

From the LIME map analysis, we can conclude that COVID-19 related cough has a unique signature. These key signatures are detected by features extracted from coughs that can be further identified and characterized by machine learning models.

### Performance parameters of model in validation phase

**Figure 4:**
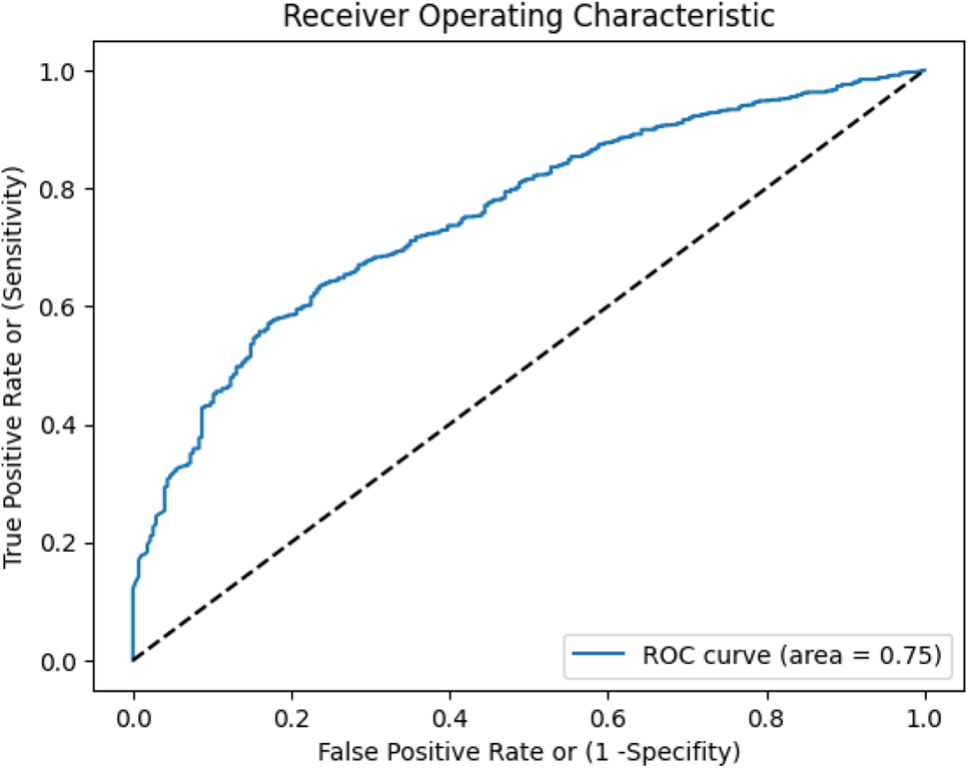
The provided ROC curve illustrates the performance of COVID-19 prediction model constructed using validation data.

Out of 234 subjects participated in the validation phase, 22 were found to be COVID-19 positive and 211 COVID-19 negative by standard diagnostic methods such as RT-PCR. Results of 1 subject remained inconclusive, hence didn’t consider that datapoint. Confusion matrix for validation phase of the model is illustrated in Table 7, where the row represents the actual label, and the column represents predicted label. An accuracy of 75.54% with 95.45% sensitivity and 73.46% specificity was achieved for the Validation phase (Table 8). Also, an AUC (Area Under the ROC Curve) of 0.75 (Fig. 4) was achieved in this phase.

**Table 7:** Confusion matrix for the validation phase. The values represented in the table are the subject number.

		Actual values (ground truths)		Total
		COVID likely—YES	COVID likely—NO	
Predicted values	COVID likely—YES	21 (TP)	56 (FP)	77
	COVID likely—NO	1 (FN)	155 (TN)	156
	Total	22	211	233

**Table 8:** Performance metrics of the validation phase.

Statistic	Value	95% CI
Sensitivity	95.45%	75.16% to 99.88%
Specificity	73.46%	69.96% to 79.29%
Positive likelihood ratio	3.60	2.82 to 4.58
Negative likelihood ratio	0.06	0.01 to 0.42
Disease prevalence	9.44%	6.01% to 13.95%
Positive predicate value	27.27%	22.74% to 32.33%
Negative predicate value	99.36%	95.80% to 99.91%
Accuracy	75.54%	69.50% to 80.91%

### Model output in the pilot phase

A total of 183 patients were recruited for the pilot testing phase. Out of these 183 subjects, model was able to identify 82 subjects as having a likely presence of SARS-CoV-2. Out of these 82 subjects, 58 truly turned out to be COVID-19 positive with a Positive predictive value (PPV) of 70.73%. The confusion matrix for this phase is enlisted in Table 9.

**Table 9:** Confusion matrix for the pilot phase. The values represented in the table are the subject number.

		Actual values (ground truths)		Total
		COVID likely—YES	COVID likely—NO	
Predicted values	COVID likely—YES	58 (TP)	24 (FP)	82
	COVID likely—NO	47 (FN)	48 (TN)	95
	Total	105	72	177

The screening of COVID-19 patients by the model is time saving as compared to the currently used traditional procedures. Additionally, our model does not need any trained professional; a community healthcare worker can also perform the screening. The technician did not need any specialised equipment or supplies. The only prerequisites to complete the exam are a smartphone and a stable internet connection.

## Discussion

The sense of urgency in comprehending, detecting, and finding a cure for SARS-CoV-2 has resulted in expedited scientific progress. This progress has been instrumental in reducing disease transmission through the acquisition of crucial knowledge, the implementation of preventive measures, and the advancement of effective treatments^32^. However, the excessive cost of rapid screening diagnostic kits as well as multiple genetic variants of the virus poses a major hindrance in conducting large-scale screening operations^33^. Various researchers across the globe are working to find a cost-effective solution to keep a check on the rapidly mutating virus^34,35^.

Machine learning (ML) has immense potential for accurate and rapid detection of various medical conditions^36^, including COVID-19, using computed tomography (CT), chest radiography (CXR), and even coughing pattern^37,38^. Specifically the information in the cough has been previously applied in the diagnosis and prediction of various diseases, including lung cancer, bronchitis, pneumonia, COPD, and asthma^39,40^.

In this study, we developed the model by merging the final output layers of the two separate models i.e., the tabular model (training input: primary and secondary features) and CNN model (training input: MFCC spectrograms). Hence, our model provides better prediction outcome as compared to the either logical repression or CNN model used alone by other researchers^19,38,41–44^. We conducted the derivation phase, validation phase and pilot screening on a comparatively large cohort, whereas previous studies were performed on either smaller scale or crowdsource datasets which are highly unreliable^38^. In a prior study the authors extracted the MFCCs from cough recordings and fed them into a pretrained CNN model, which resulted in an AUC (Area Under the ROC Curve) of 97% with a sensitivity and a specificity of 94.2%^17^. Another AI-based COVID-19 cough classifier study includes the analysis of cough recorded over a smartphone, which was able to distinguish COVID-19 positive cases from both COVID-19 negative and healthy coughs. An AUC (Area Under the ROC Curve)of 98% was achieved using the Resnet50 classifier to discriminate between COVID-19 positive and healthy coughs, while to differentiate between COVID-19 positive and negative coughs an LSTM classifier was used with an AUC of 94%^19^. In one of the studies both coughs and breathing sounds were used to identify how distinct COVID-19 sounds were as compared to asthma patients or healthy individuals. They highlighted that how a simple binary machine-learning classifier can distinguish COVID-19 cough sounds from healthy subjects by achieving an overall AUC of above 80%^41^. A recent study made use of an ensemble-based multi-criteria decision-making (MCDM) method for detecting COVID-19 from cough sound data and achieved an AUC of 95%^38^.

Our model achieved an accuracy of 75.54% with 95.45% sensitivity and 73.46% specificity in the clinical validation phase. The pilot testing was undertaken in a real primary care setting to test the accuracy of the tool. Upon deployment as a screening and triaging tool prior to molecular testing, this model was proven statistically effective in identifying high-risk patients for confirmatory testing. In the pilot phase also, the model achieved a positive prediction value of 70.73% in a clinical setup at a tertiary care hospital.

Swaasa has been specifically developed as a screening tool with the primary objective of assisting healthcare professionals in identifying individuals who may potentially have COVID-19. By harnessing the capabilities of our advanced model, our aim is to significantly improve the efficiency and precision of the screening process. This, in turn, facilitates the prioritization of individuals for further evaluation, leading to more targeted and timely interventions for those suspected of being infected with COVID-19. Our model serves as a valuable resource in enhancing the accuracy and effectiveness of COVID-19 screening efforts.

## Data Availability

Due to the nature of this research, participants of this study did not agree for their data to be shared publicly. However, the detailed analysis can be shared by the author “NRS” upon reasonable request.
